# Ergot in Dysentery

**Published:** 1874-11

**Authors:** 


Ergot in Dysentery.—A French physician claims to have succeeded in the treatment of epidemic dysentery by the use of ergot. He gave seven or eight grains every four hours; the cure being effected in ordinary cases in two or three days. After a few doses, constipation is produced, which lasts three or four days. Ergotin has the same effect.—*Ex.*

